# Transcatheter Aortic Valve Replacement in Patients with Aortic Stenosis Having Coronary Cusp Fusion versus Mixed Cusp Fusion Nonraphe Bicuspid Aortic Valve

**DOI:** 10.1155/2019/7348964

**Published:** 2019-11-03

**Authors:** Wen-hua Lei, Yan-biao Liao, Zi-jie Wang, Yuan-weixiang Ou, Jiay-yu Tsauo, Yi-jian Li, Tian-yuan Xiong, Zhen-gang Zhao, Xin Wei, Wei Meng, Yuan Feng, Mao Chen

**Affiliations:** ^1^Department of Cardiology, West China Hospital, Sichuan University, 37 Guoxue Street, Chengdu 610041, China; ^2^Department of Cardiovascular Surgery, West China Hospital, Sichuan University, 37 Guoxue Street, Chengdu 610041, China

## Abstract

**Objectives:**

We aimed to assess the procedural and clinical results of transcatheter aortic valve replacement (TAVR) for nonraphe bicuspid aortic stenosis (AS) with coronary vs mixed cusp fusion.

**Background:**

It remains unclear whether cusp fusion morphology affects TAVR outcomes in patients with nonraphe bicuspid AS.

**Methods:**

This retrospective study enrolled consecutive patients with severe symptomatic AS and type-0 bicuspid aortic valve, who underwent TAVR at our institution between 2012 and 2017. TAVR outcomes were defined based on the Valve Academic Research Consortium-2 recommendations.

**Results:**

Compared to patients with mixed cusp fusion (44/71), those with coronary cusp fusion (27/71) had a larger ellipticity index for the aortic annulus (21.9% ± 9.0% vs 15.6% ± 9.3%, *p*=0.007) and increased left ventricular outflow tract obstruction (31.1% ± 9.4% vs 26.9% ± 7.5%, *p*=0.04) but comparable rates of second valve implantation (15.9% vs 14.8%), mild paravalvular leakage (PVL, 38.5% vs 30.2%), permanent pacemaker implantation (PPM, 25.9% vs 15.9%), and 30-day mortality (7.4% vs 6.8%). Use of a first-generation transcatheter heart valve was associated with higher risk for mild PVL (odds ratio (OR) = 4.37; 95% confidence interval (95% CI) = 1.14–16.75; *p*=0.03) but not PPM (OR = 0.77; 95% CI = 0.22–2.62; *p*=0.67), whereas a larger oversizing ratio tended to be associated with a higher PPM rate (OR = 1.49; 95% CI = 0.46–4.86; *p*=0.51) but lower incidence of mild PVL (OR = 0.51; 95% CI = 0.19–1.35; *p*=0.17).

**Conclusions:**

In AS patients with type-0 bicuspid valves, cusp fusion morphology does not affect the procedural or clinical results of TAVR. Use of second-generation transcatheter heart valves may provide more favorable results in such patients. This trial is registered with NCT01683474.

## 1. Introduction

With the development and increasing evidence of transcatheter aortic valve replacement (TAVR), TAVR has already evolved as a well-established option for symptomatic aortic stenosis (AS) in patients who are deemed at intermediate risk [1, 2]. Recent evidence suggests that TAVR provides good results in younger AS patients even at low risk [3–5]. While these findings are promising, extending the TAVR indication to younger AS patients is expected to increase the proportion of TAVR recipients with bicuspid morphology [6]. Several studies have reported the feasibility and efficacy of TAVR in AS patients with bicuspid aortic valve, but the results were heterogeneous [7–12]. In an attempt to facilitate comparison, Jilaihawi et al. described a TAVR-focused classification of bicuspid valves based on leaflet morphology and orientation, wherein bicommissural valves with no raphe are equivalent to type-0 bicuspid valves defined by Sievers and Schmidtke [9, 13].

The proportion of AS patients with bicuspid morphology treated via TAVR differs between Asian and Western countries [9]. Among Asian AS patients with bicuspid valve indicated for TAVR, those with type-0 morphology account for more than 50%. According to Jilaihawi et al., type-0 bicuspid morphology in patients with AS can be classified according to whether only the coronary cusps are fused (coronary cusp fusion) or whether fusion involves the noncoronary cusp (mixed cusp fusion) [9]. To date, none studies have examined the effect of cusp fusion morphology on the results of TAVR in patients with type-0 bicuspid aortic valve morphology. Thus, in the present study, we aimed to explore the effect of coronary vs mixed cusp fusion on the procedural and clinical results of AS patients with type-0 bicuspid morphology who undergo TAVR with a first- or second-generation transcatheter heart valve (THV).

## 2. Materials and Methods

### 2.1. Patients

Consecutive patients with severe symptomatic AS who underwent TAVR at our institution between April 2012 and February 2017 were recruited in the present study. The indication for TAVR was discussed at length by our TAVR heart team. All patients provided informed consent for undergoing the recommended procedures. This retrospective study was approved by the institutional review board of our hospital.

### 2.2. Definitions

**Figure 1:**
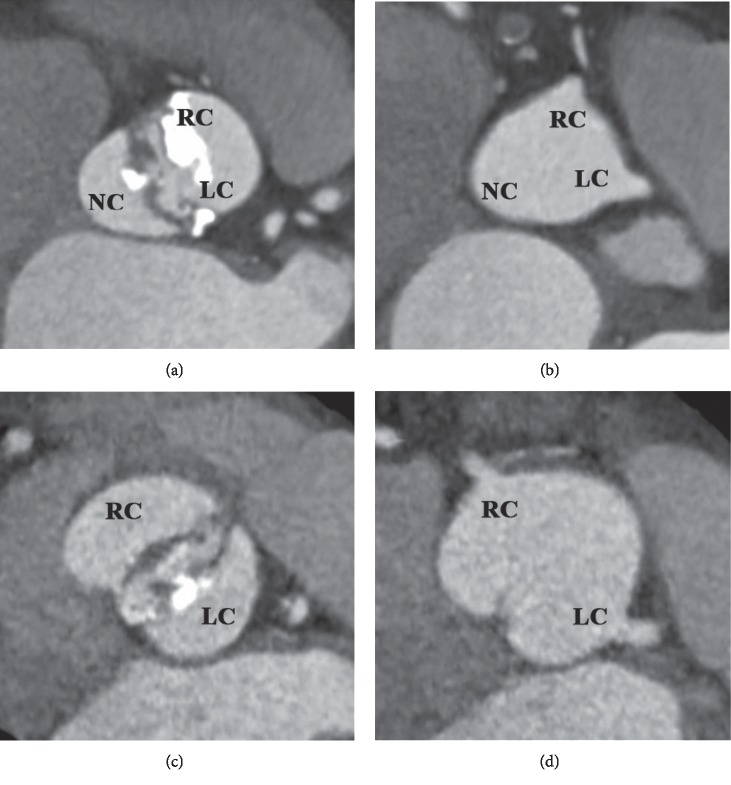
Definition of cusp fusion in type-0 bicuspid aortic stenosis: coronary cusp fusion morphology (a, b); mixed cusp fusion morphology (c, d). LC, left coronary cusp; NC, noncoronary cusp; RC, right coronary cusp.

Type-0 bicuspid aortic valve morphology was defined according to the classification proposed by Sievers and Schmidtke [13]. Type-0 bicuspid valves were further classified according to cusp fusion morphology into coronary and mixed cusp fusion subtypes, as proposed by Jilaihawi et al. (Figure 1) [9].

### 2.3. Pre-TAVR Aortic Root Evaluation

The methods are in accordance with our previous study reported by Liao et al. [14]. Briefly, to choose the appropriate valve size, pre-TAVR aortic root evaluation was performed using multisliced computed tomography (MSCT). The dimensions of aortic root were evaluated on the MSCT scan using the OsiriX DICOM viewer software (OsiriX Foundation, Geneva, Switzerland), whereas the volume of aortic root calcification was calculated using FluoroCT 3.0 (Circle Cardiovascular Imaging Inc., Calgary, Canada) [14]. A plane which passes the two lowest points of bicuspid valvular cuspus and below all the points of bicuspid valvular cuspus was regarded as annulus. The grade of calcification of the left ventricular outflow tract (LVOT) was evaluated semiquantitatively as follows: mild, one calcification nodule extending <5 mm in any direction and covering <10% of the LVOT perimeter; moderate, two calcification nodules or one extending >5 mm in any direction or covering >10% of the LVOT perimeter; and severe, multiple calcification nodules of single focus extending >1 cm in length or covering >20% of the LVOT perimeter [15]. The ellipticity index of the aortic root was calculated as (1 − short/long axis) × 100.

For self-expandable valves, oversizing was calculated as (prosthesis nominal perimeter/MSCT-derived annular perimeter − 1) × 100. For Lotus valves, oversizing was calculated as (prosthesis nominal area/MSCT-derived annular area − 1) × 100.

### 2.4. TAVR Procedure

Full details regarding the TAVR procedure were provided elsewhere [16]. The TAVR heart team determined the most suitable approach route. Four types of THVs were used in this series, namely, Medtronic CoreValve (Medtronic, USA) and Venus-A (Venus Medtech, China) as first-generation valves; VitaFlow (MicroPort, China) and Lotus (Boston Scientific, USA) as second-generation valves. The Medtronic CoreValve, Venus-A, and VitaFlow are self-expandable valves. The Venus-A valve was described in detail in our previous study [16]. VitaFlow consists of a nitinol stent with low density, large cells, a bovine pericardial leaflet, and a prolonged polyethylene glycol terephthalate skirt extending beyond the inflow segment to improve sealing and reduce paravalvular leakage (PVL). Balloon pre- and postdilation were performed at the operator's discretion. The method for sizing the THV in bicuspid morphology was reported in our previous study [14]. Briefly, balloon sizing was used to choose the proper size of THV. The size of balloon was chosen which equals the minor diameter of annulus measured on MSCT. If the chosen balloon behaved appropriately, that was no contrast leaking to the left ventricle, and coronary arteries were patent on angiography, the average diameter of the annulus would be determined as balloon size plus 3 mm. This average diameter would then be used to choose the valve size according to the sizing chart provided by the manufacturer. Otherwise, if contrast leakage did exist, a one size bigger valve than selected by the average diameter will be chosen [14]. The degree of post-TAVR PVL was evaluated on echocardiography and classified as none/trace (grade 0), mild (grade 1), moderate (grade 2), and severe (grade 3). In patients with moderate or severe aortic regurgitation unresponsive to postdilation, implantation of an additional valve was considered.

### 2.5. Follow-Up and Outcomes

Patients were followed primarily by office visits and telephone interviews. Clinical results were defined according to the Valve Academic Research Consortium-2 (VARC-2) recommendations [17].

### 2.6. Statistical Analysis

Continuous variables described as mean ± standard deviation were compared using the unpaired Student's *t*-test, whereas those described as median (interquartile range) were compared using the Mann–Whitney nonparametric test. Categorical variables were expressed as percentages and compared using the chi-squared test or Fisher's exact test, as appropriate. Relative associations were described in terms of odds ratios (ORs) with 95% confidence intervals (CIs) and *p* values. All statistical analyses were performed using SPSS version 20.0 (IBM Inc., Armonk, NY, USA) with two-tailed significance set at 0.05.

## 3. Results

### 3.1. Patient Baseline Characteristics

A total of 215 patients underwent TAVR at our institution between April 2012 and February 2017. Finally, 71 (33%) AS patients with type-0 bicuspid aortic valve (coronary cusp fusion, *n* = 27; mixed cusp fusion, *n* = 44) and an average Society of Thoracic Surgeons score of 7.0% were included in the present study (Supplementary Information [Supplementary-material supplementary-material-1]). The median follow-up duration was 547 days (interquartile range, 357–1079 days). No significant differences were observed between the two groups regarding the proportion of male patients or the prevalence of comorbidities (Table 1).

### 3.2. Pre-TAVR Aortic Root Dimensions

Patients with coronary cusp fusion had more elliptical annulus and LVOT than those patients with mixed cusp fusion. Other parameters of aortic root morphology were comparable between the two groups (Supporting Information [Supplementary-material supplementary-material-1]).

### 3.3. Pre-TAVR Echocardiographic Characteristics

There was no significant difference between patients with coronary and mixed cusp fusion regarding the maximum velocity of the aortic jet, the mean paravalvular gradient, or other relevant echocardiographic characteristics of pre-TAVR (Supporting Information [Supplementary-material supplementary-material-1]).

### 3.4. Procedural and Clinical Results

The post-TAVR results are summarized in Tables 2 and 3. All patients underwent TAVR via the transfemoral approach. More than 90% of patients received predilation before valve implantation. The preference for certain THV types did not differ between the two groups, but patients with coronary cusp fusion tended to receive larger valves. In addition, the two groups did not differ in terms of the rate of second valve implantation (*p*=1.0). Nearly, 40% of patients received postdilation after valve implantation (*p*=0.8, Table 2).

Patients with coronary and mixed cusp fusion had similar incidence of left bundle branch block (*p*=0.59), permanent pacemaker implantation (*p*=0.36), major vascular complications (*p*=1.0), major bleeding (*p*=1.0), and stroke (*p*=1.0). No annular rupture or coronary obstruction occurred. Finally, the coronary and mixed cusp fusion morphologies were associated with comparable 30-day (*p*=1.0) and 1-year (*p*=0.67) all-cause mortality (Table 3).

### 3.5. First-Generation vs Second-Generation Valves

Compared to using second-generation THVs, using first-generation THVs was associated with higher risk for mild PVL (OR, 4.37; 95% CI, 1.14–16.75; *p*=0.03) and postdilation (OR, 3.98; 95% CI, 1.18–13.48; *p*=0.03), but similar incidence of LBBB (OR, 2.79; 95% CI, 0.2–10.9; *p*=0.14), PPM (OR, 0.77; 95% CI, 0.22–2.62; *p*=0.67), and second valve implantation (OR, 2.25; 95% CI, 0.44–11.41; *p*=0.33) (Supplementary Information [Supplementary-material supplementary-material-1]).

### 3.6. Larger vs Smaller Oversizing Ratio

Upon examining the incidence of adverse events according to oversizing ratio dichotomized as above and below the median value for each valve type (Supplementary Information [Supplementary-material supplementary-material-1]), we found that larger oversizing ratio tended to be associated with higher incidence of LBBB (OR, 2.64; 95% CI, 0.86–8.13; *p*=0.09) and PPM (OR, 1.49; 95% CI, 0.46–4.86; *p*=0.51), but lower incidence of mild PVL (OR, 0.51; 95% CI, 0.19–1.35; *p*=0.17). However, patients with larger oversizing ratio had significantly lower risk for second valve implantation (OR, 0.18; 95% CI, 0.04–0.91; *p*=0.04).

### 3.7. Post-TAVR Echocardiographic Results

No significant difference between patients with coronary and mixed cusp fusion was observed regarding the maximum velocity of the aortic jet (*p*=0.81) or mean paravalvular gradient (*p*=0.57). No patient had more than moderate PVL post-TAVR because we always inserted a second valve to treat more than moderate PVL irresponsive to postdilation. The overall incidence of mild PVL was >30% and did not differ between patients with coronary cusp fusion and those with mixed cusp fusion (*p*=0.6; Table 4).

## 4. Discussion

The major findings of the present study are as follows: (i) compared to mixed cusp fusion morphology, coronary cusp fusion morphology in AS patients with type-0 bicuspid aortic valve is characterized by more elliptical annulus and LVOT; (ii) in AS patients with type-0 bicuspid aortic valve, the procedural results, clinical results, and hemodynamics at discharge post-TAVR were not affected by the cusp fusion morphology; (iii) compared to using second-generation THVs, using first-generation THVs was associated with higher risk for postdilation and mild PVL but similar incidence of LBBB, PPM, and second valve implantation; (iv) large oversizing ratio was associated with lower risk for second valve implantation.

Bicuspid aortic valve disease is the most common congenital cardiac abnormality, affecting 0.5% to 2.0% of the general population [18]. In patients with bicuspid aortic valve, AS is still regarded as an off-label indication for TAVR because such patients typically exhibit severely calcified leaflets, elliptic annulus, and enlarged ascending aorta [19]. Nevertheless, TAVR has achieved encouraging procedural and clinical outcomes in AS patients with bicuspid aortic valve, with new-generation devices providing outcomes comparable to those noted in AS patients with tricuspid valve [12, 20, 21]. However, the TAVR results in AS patients with bicuspid aortic valve have been heterogeneous, which may be related to morphological variation [7–9]. To clarify this aspect, Jilaihawi et al. proposed a TAVR-focused classification of bicuspid aortic valve morphology, based on leaflet morphology and orientation [9]. Interestingly, the prevalence of bicommissural bicuspid aortic valve differed between Asian and Western countries. Specifically, while Asian AS patients with bicuspid valve often exhibit bicommissural, nonraphe morphology similar to the type-0 bicuspid morphology described by Sievers and Schmidtke, Western patients typically have bicommissural raphe type morphology [9]. None studies have compared the effect of coronary vs mixed cusp fusion on the results of TAVR in AS patients with type-0 bicuspid aortic valve.

In the present study, we found that, compared to mixed cusp fusion morphology, coronary cusp fusion morphology was characterized by more elliptical annulus and LVOT, but that the rates of annular rupture, second valve implantation, LBBB, PPM, mild PVL, 30-day mortality, and 1-year mortality did not differ between the two groups. These findings suggest that further classification of type-0 bicuspid aortic valves based on leaflet orientation may not affect the utility of TAVR for AS.

Importantly, we found that, in AS patients with type-0 bicuspid valve, the outcomes of TAVR were acceptable (annular rupture, 0%; coronary obstruction, 0%; stroke, 4.2%; and 30-day all-cause mortality, 7%) and comparable to those previously reported in AS patients with tricuspid valve [20, 22]. Encouragingly, the rate of mild PVL after TAVR for AS in patients with type-0 bicuspid valve was more favorable for second-generation THVs than for first-generation THVs (13.6% vs 40.8%, *p*=0.03), which is in line with previous observations [10, 12]. However, the rate of PPM was comparable in patients with first-generation THVs and those with second-generation THVs. This was previously reported for balloon-expandable valves known to be associated with lower risk for PPM in AS patients with tricuspid valve [10, 12].

In this series, the median oversizing ratio was 13.2% for CoreValve/Venus-A, 12.8% for Lotus, and 1.9% for VitaFlow. We found that oversizing ratios above the median tended to be associated with higher incidence of LBBB and PPM but lower incidence of mild PVL and second valve implantation ([Supplementary-material supplementary-material-1]). The incidence of implanting second valve in the present study was 15.5%, which was related to the smaller valve we choose to treat type-0 bicuspid valve. When we chose a larger valve, the incidence of implanting second valve in patients with larger oversizing ratio was only 5.5% ([Supplementary-material supplementary-material-1]). In a study of 51 AS patients with bicuspid valve who underwent TAVR with the SAPIEN 3 valve (mean area-based oversizing ratio: 13.5%), Perlman et al. reported the incidence of mild PVL at 37.2% and the rate of PPM at 23.5% [10]. In a study of 301 AS patients with bicuspid valve who underwent TAVR with Sapien XT, CoreValve, Sapien 3, or Lotus valves (average area-based oversizing ratio: 19.6%, 33.1%, 12.9%, and 8.4%, respectively), Yoon et al. reported the incidence of mild PVL at 36.8%, 39.3%, 15.4%, and 18.2%, respectively, and the rate of PPM at 9.2%, 16.1%, 17.6%, and 9.1%, respectively [11]. These heterogeneous results of TAVR in patients with bicuspid valve may be related to the different prevalence of specific morphologic subtypes and the use of different oversizing ratios. Thus, further studies are warranted to determine the optimal oversizing ratio for TAVR with various THVs in patients with specific bicuspid valve subtypes. In this context, it may be useful to apply supraannular sizing, which takes into consideration supraannular structures including leaflet calcification and intercommissural distance for determining the appropriate valve size. Moreover, as implantation depth correlates with PPM rate after TAVR for AS in patients with bicuspid valve, [9] it may be particularly important to achieve the optimal implantation depth in this patient population.

Several limitations should be considered when interpreting the results of the present study. First, this study reflects the clinical experience in a single center and had an observational design; thus, although the two groups were comparable in terms of baseline characteristics, selection bias could not be excluded. Second, we did not conduct propensityscore matching analysis because the sample was small and because there was no significant difference between the groups regarding baseline characteristics. Finally, we reviewed TAVI procedures performed between April 2012 and February 2017, during which the experience of our TAVR heart team increased. Thus, the accumulation of clinical experience should be taken into consideration when interpreting the conclusions regarding the comparison between first- and second-generation devices.

## 5. Conclusion

In AS patients with type-0 bicuspid aortic valve, the results of TAVR were not affected by cusp fusion morphology (coronary vs mixed). Second-generation THVs may provide more favorable outcomes in AS patients with type-0 bicuspid valve, but further studies are warranted to determine the optimal oversizing ratio for different THVs.

## Figures and Tables

**Table 1:** Baseline characteristics.

Characteristic	Total (*N* = 71)	Mixed fusion (*N* = 44)	Coronary fusion (*N* = 27)	*p* value
Age (years)	71.9 ± 5.8	71.5 ± 6.1	72.6 ± 5.4	0.44
Male sex	32 (45.1%)	20 (45.5%)	12 (44.4%)	1.0
Body mass index (kg/m^2^)	22.3 ± 3.4	22.2 ± 3.5	22.5 ± 3.2	0.80
Body surface area (m^2^)	1.65 ± 0.13	1.65 ± 0.14	1.64 ± 0.12	0.84
STS score (%)	7.0 ± 3.6	6.9 ± 4.0	7.2 ± 3.0	0.76
NYHA class III/IV	63 (88.7%)	39 (88.6%)	24 (88.9%)	1.0
Creatinine (mg/dL)	0.9 (0.8–1.1)	0.9 (0.7–1.1)	0.93 (0.86–1.1)	0.13
Hypertension	32 (45.1%)	18 (40.9%)	14 (51.9%)	0.46
Diabetes	14 (19.7%)	8 (18.2%)	6 (22.2%)	0.76
Chronic lung disease	37 (52.1%)	22 (50%)	15 (55.6%)	0.81
Coronary artery disease	25 (35.2%)	15 (34.1%)	10 (37.0%)	1.0
Peripheral vascular disease	36 (50.7%)	23 (52.3%)	13 (48.1%)	0.81
Cerebral vascular disease	16 (22.5%)	11 (25%)	5 (18.5%)	0.57
Chronic kidney disease	6 (8.5%)	3 (6.8%)	3 (11.1%)	0.67
Previous myocardial infarction	2 (2.8%)	1 (2.3%)	1 (3.7%)	1.0
History of permanent pacemaker	3 (4.2%)	2 (4.5%)	1 (3.7%)	1.0
Cancer	3 (4.2%)	3 (6.8%)	0	0.28

The patients were stratified according to the type of cusp fusion. Data are shown as mean ± standard deviation, frequency (percentage), or median (range). NYHA, New York Heart Association; STS, Society of Thoracic Surgeons.

**Table 2:** Procedural characteristics.

Characteristic	Total (*N* = 71)	Mixed fusion (*N* = 44)	Coronary fusion (*N* = 27)	*p* value
Transfemoral approach	71 (100%)	44 (100%)	27 (100%)	1.0
Local anesthesia	9 (12.7%)	6 (13.6%)	3 (11.1%)	0.52
Predilation	66 (93.0%)	41 (93.2%)	25 (92.6%)	1.0
Oversizing ratio (%)^*∗*^	12.2 ± 11.3	12.5 ± 11.7	11.7 ± 10.7	0.79
Implantation depth (mm)^†^	6.5 ± 3.8	6.8 ± 3.6	5.9 ± 4.1	0.37
Transcatheter heart valve type				0.83
Medtronic CoreValve	16 (22.5%)	11 (25%)	5 (18.5%)	0.57
Venus-A	33 (46.5%)	21 (47.7%)	12 (44.4%)	0.81
VitaFlow	6 (8.5%)	3 (6.8%)	3 (11.1%)	0.67
Lotus	16 (22.5%)	9 (20.5%)	7 (25.9%)	0.77
Mean valve diameter (mm)	26.1 ± 2.2	25.9 ± 1.7	26.7 ± 2.9	0.152
Medtronic CoreValve/Venus-A				**0.005**
23	3 (4.2%)	2 (4.5%)	1 (3.7%)	1.0
26	30 (42.3%)	24 (54.5%)	6 (22.2%)	**0.013**
29	12 (16.9%)	6 (13.6%)	6 (22.2%)	0.30
31/32	4 (5.6%)	0	4 (14.8%)	**0.011**
VitaFlow				0.40
24	4 (5.6%)	1 (2.3%)	3 (11.1%)	
27	2 (2.8%)	2 (4.5%)	0	
Lotus valve				1.0
23	7 (9.9%)	4 (9.1%)	3 (11.1%)	
25	9 (12.7%)	5 (11.4%)	4 (14.8%)	
Need for a second valve	11 (15.5%)	7 (15.9%)	4 (14.8%)	1.0
Postdilation	28 (39.4%)	17 (38.6%)	11 (40.7%)	0.80

The patients were stratified according to the type of cusp fusion. Data are shown as mean ± standard deviation or frequency (percentage). ^*∗*^Oversizing ratio calculated based on valve perimeter for self-expandable valves and based on valve area for Lotus valves. ^†^Implantation depth defined as the distance between the lowest point of the noncoronary sinus and the corresponding inflow part of the frame.

**Table 3:** Clinical results of transcatheter aortic valve replacement.

Outcome	Total (*N* = 71)	Mixed fusion (*N* = 44)	Coronary fusion (*N* = 27)	*p* value
Left bundle branch block	19 (26.8%)	13 (29.5%)	6 (22.2%)	0.59
Permanent pacemaker implantation	14 (19.7%)	7 (15.9%)	7 (25.9%)	0.36
Annulus rupture	0	0	0	—
Vascular complication				0.74
None	56 (78.9%)	36 (81.8%)	20 (74.1%)	0.55
Minor	10 (14.1%)	5 (11.4%)	5 (18.5%)	0.49
Major	5 (7.0%)	3 (6.8%)	2 (7.4%)	1.0
Bleeding complication				0.27
None	49 (69.0%)	33 (75%)	16 (59.3%)	0.19
Minor	14 (19.7%)	6 (13.6%)	8 (29.6%)	0.13
Major	8 (11.3%)	5 (11.4%)	3 (11.1%)	1.0
Coronary obstruction	0	0	0	—
Cerebrovascular complication				0.72
None	67 (94.4%)	42 (95.5%)	25 (92.6%)	0.63
Transient ischemic attack	1 (1.4%)	0	1 (3.7%)	0.38
Stroke	3 (4.2%)	2 (4.5%)	1 (3.7%)	1.0
30-day mortality	5 (7.0%)	3 (6.8%)	2 (7.4%)	1.0
1-year mortality	6 (8.5%)	3 (6.8%)	3 (11.1%)	0.67

The patients were stratified according to the type of cusp fusion. Data are shown as frequency (percentage).

**Table 4:** Echocardiographic characteristics at discharge after transcatheter aortic valve replacement.

Characteristic	Total (*N* = 69)	Mixed fusion (*N* = 43)	Coronary fusion (*N* = 26)	*p* value
*V*_max_ (m/s)	2.5 (2.1–2.9)	2.5 (2.2–2.9)	2.5 (2.1–2.9)	0.81
PG_mean_ (mmHg)	15.6 ± 6.7	15.9 ± 6.5	15.0 ± 7.0	0.57
LVEF (%)	61.0 (54.0–66.0)	62.0 (57.0–66.0)	59.5 (45.0–66.5)	0.53
Paravalvular leakage				0.6
None/trace	46 (66.6%)	30 (69.8%)	16 (61.5%)	0.6
Mild	23 (33.3%)	13 (30.2%)	10 (38.5%)	0.6
Moderate to severe	0	0	0	—
Mitral regurgitation				0.13
None/trace	34 (49.3%)	25 (58.1%)	9 (34.6%)	0.08
Mild	32 (46.4%)	17 (39.5%)	15 (57.7%)	0.21
Moderate to severe	3 (4.3%)	1 (2.3%)	2 (7.7%)	0.56
Tricuspid regurgitation				0.75
None/trace	47 (68.1%)	30 (69.8%)	17 (65.4%)	0.79
Mild	21 (30.4%)	12 (27.9%)	9 (34.6%)	0.60
Moderate to severe	1 (1.4%)	1 (2.3%)	0	1.0

The patients were stratified according to the type of cusp fusion. Data are shown as median (range), mean ± standard deviation, or frequency (percentage). LVEF, left ventricular ejection fraction; PG_mean_, mean paravalvular gradient; *V*_max_, maximum velocity of the aortic jet.

## Data Availability

The statistical data used to support the findings of this study are available from the corresponding author upon request.

## Supplementary Materials

Supplementary Materials Supplementary Information Figure S1: study flow chart. Supplementary Information Figure S2: procedural and clinical results of transcatheter aortic valve replacement for type-0 bicuspid aortic stenosis. Supplementary Information Figure S3: impact of oversizing ratio on the outcomes of transcatheter aortic valve replacement. Supplementary Table 1: baseline imaging characteristics on multisliced computed tomography. Supplementary Table 2: baseline echocardiographic characteristics.
