# Supplementary material for: Transcatheter Aortic Valve Replacement in Patients with Aortic Stenosis Having Coronary Cusp Fusion versus Mixed Cusp Fusion Nonraphe Bicuspid Aortic Valve
Source: J Interv Cardiol. 2019 Nov 3;2019:7348964. doi: 10.1155/2019/7348964 (PMC6874966; doi:10.1155/2019/7348964)
Supplement: Supplementary Materials — Supplementary Information Figure S1: study flow chart. Supplementary Information Figure S2: procedural and clinical results of transcatheter aortic valve replacement for type-0 bicuspid aortic stenosis. Supplementary Information Figure S3: impact of oversizing ratio on the outcomes of transcatheter aortic valve replacement. Supplementary Table 1: baseline imaging characteristics on multisliced computed tomography. Supplementary Table 2: baseline echocardiographic characteristics. [file 7348964.f1.pdf]

Supplementary Materials

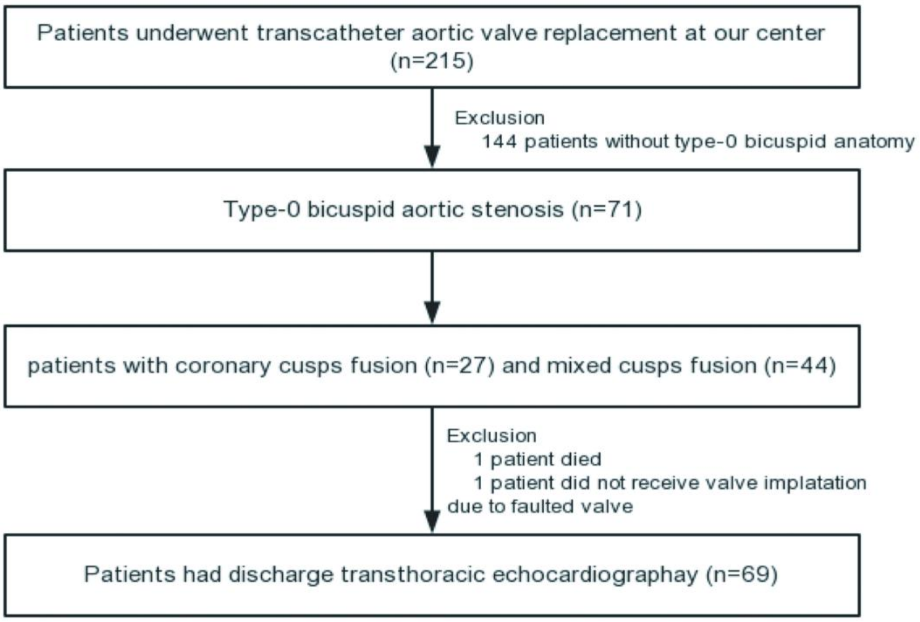

Supplementary Information Figure S1. Study flow chart

Among the 215 patients who underwent transcatheter aortic valve replacement at our center, 71 had type-0 bicuspid aortic stenosis. Of these, 27 and 44 patients had coronary and mixed cusp fusion, respectively.

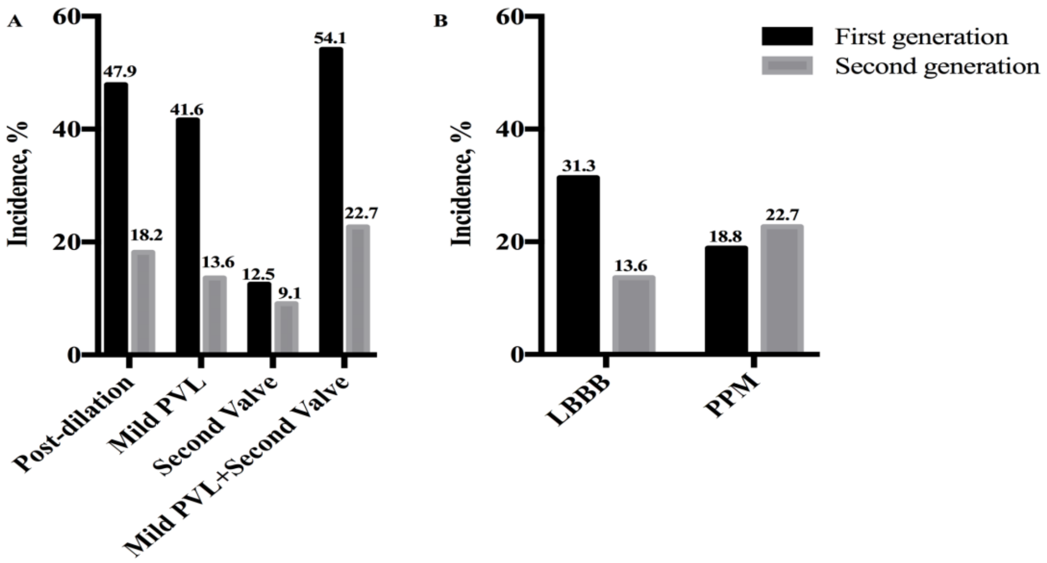

Supplementary Information Figure S2. Procedural and clinical results of transcatheter aortic valve replacement for type-0 bicuspid aortic stenosis

Data are shown for first-generation (Medtronic CoreValve/Venus-A) and second-generation valves (Lotus, VitaFlow). Procedural results (A) include the rates of post-dilation and mild PVL, second valve implantation, or both. Clinical results (B) include the rates LBBB, and PPM. LBBB, left bundle branch block; PPM, permanent pacemaker implantation; PVL, paravalvular leakage.

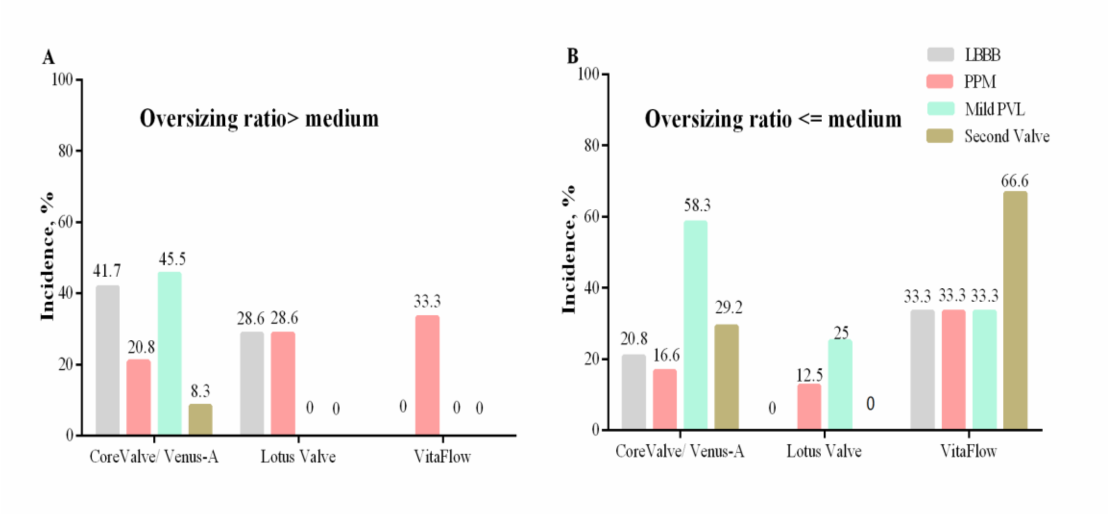

Supplementary Information Figure S3. Impact of oversizing ratio on the

outcomes of transcatheter aortic valve replacement

Outcome data were dichotomized for oversizing ratio above (A) or below (B) the median value, which was 13.2% for CoreValve/Venus-A valves, 12.8% for Lotus valves, and 1.9% for VitaFlow valves. LBBB, left bundle branch block; PPM, permanent pacemaker implantation; PVL, paravalvular leakage.

Supplemental Table 1 Baseline imaging characteristics on multi-slice computed tomography

Characteristic	Total (N=71)	Mixed fusion (N=44)	Coronary fusion (N=27)	P-valve
Annulus				
Short diameter, mm	21.2±2.9	21.3±2.8	21.1±3.2	0.81
Long diameter, mm	26.0±3.3	25.4±3.1	27.1±3.3	**0.027**
Ellipticity index, %*	18.0±9.6	15.6±9.3	21.9±9.0	**0.007**
Ellipticity index ≥20%	32 (45.1%)	17 (38.6%)	15 (55.6%)	0.22
Perimeter, mm	75.4±8.9	74.2±8.3	77.3±9.7	0.16
Area, mm^2	438.2±105.7	427.2±99.2	456.2±115.3	0.27
LVOT				
Short diameter, mm	20.3±4.0	20.6±3.6	19.8±4.7	0.40
Long diameter, mm	28.3±4.0	28.2±3.9	28.5±4.4	0.78
Ellipticity index, %*	28.5±8.5	26.9±7.5	31.1±9.4	**0.04**

Ellipticity index ≥20%	59 (83.1%)	35 (79.5%)	24 (88.9%)	0.35
Perimeter, mm	80.5±11.3	80.1±11.1	81.1±11.9	0.71
Area, mm^2	470.0±153.2	477.5±138.4	457.7±176.7	0.60
Sinus of Valsava				
Short diameter, mm	26.7±3.2	37.3±4.2	36.7±4.2	0.59
Long diameter, mm	37.1±4.2	26.3±3.3	27.3±2.9	0.20
Perimeter, mm	106.9±12.7	106.1±12.6	108.1±13.1	0.53
Area, mm^2	873.8±213.7	860.8±210.9	895.2±220.5	0.51
Intercommissural distance, mm	24.7±2.6	24.3±2.5	25.3±2.6	0.12

Supplemental Table 2 Baseline echocardiographic characteristics

Characteristics	Total (N=71)	Mixed fusion (N=44)	Coronary fusion (N=27)	P-value
V_{max}, m/s	5.3±0.7	5.3±0.7	5.2±0.8	0.34
PG_{mean}, mmHg	69.6±20.2	71.0±19.5	67.3±21.5	0.47
LVEF, %	53.3±16.2	55.0±16.4	50.5±15.8	0.26
Aortic regurgitation				0.77
None/trace	48 (67.6%)	31 (70.5%)	17 (63.0%)	0.60
Mild	18 (15.4%)	9 (20.5%)	9 (33.3%)	0.27
Moderate-to-severe	5 (7.0%)	4 (9.1%)	1 (3.7%)	0.64
Mitral regurgitation				0.67
None/trace	33 (46.5%)	21 (47.7%)	12 (44.4%)	0.81
Mild	28 (39.4%)	18 (40.9%)	10 (37.0%)	0.47

Moderate-to-severe	10 (14.1%)	5 (11.4%)	5 (18.5%)	0.49
Tricuspid regurgitation				0.62
None/trace	46 (64.8%)	30 (68.2%)	16 (59.3%)	0.61
Mild	22 (31.0%)	13 (29.5%)	9 (33.3%)	0.80
Moderate-to-severe	3 (4.2%)	1 (2.3%)	2 (7.4%)	0.55

The patients were stratified according to the type of cusp fusion. Data are shown as mean ± standard deviation or frequency (percentage). LVEF, left ventricular ejection fraction; PG_{mean}, mean paravalvular gradient; V_{max}, maximum velocity of the aortic jet。
